# Rhizobium acaciae sp. nov., a new nitrogen-fixing symbiovar isolated from root nodules of Acacia saligna in Tunisia

**DOI:** 10.1099/ijsem.0.005900

**Published:** 2023-05-18

**Authors:** Jihed Hsouna, Houda Ilahi, Jia-Cheng Han, Takwa Gritli, Walid Ellouze, Xiao Xia Zhang, Maroua Mansouri, Praveen Rahi, Mustapha Missbah El Idrissi, Mouad Lamrabet, M'hamed Oubla, Pierre Emmanuel Courty, Daniel Wipf, James T. Tambong, Bacem Mnasri

**Affiliations:** 1 Laboratory of Legumes and Sustainable Agrosystems, Centre of Biotechnology of Borj-Cédria, BP 901 Hammam-lif 2050, Tunisia; 2 Agricultural Cultural Collection of China, Institute of Agricultural Resources and Regional Planning, Chinese Academy of Agricultural Sciences, Beijing 100080, PR China; 3 Agriculture and Agri-Food Canada, 4902 Victoria Avenue North, Vineland Station, Ontario, L0R 2E0, Canada; 4 Institut Pasteur, Université Paris Cité, Biological Resource Center of Institut Pasteur (CRBIP), Paris, France; 5 Faculty of Sciences, Centre de Biotechnologies Végétale et Microbienne, Biodiversité et Environnement, Mohammed V University in Rabat, Rabat, Morocco; 6 Faculty of Science, Materials and Nanomaterials for Photovoltaic Conversion and Electrochemical Storage, Mohammed V University in Rabat, Rabat, Morocco; 7 Agroécologie, Institut Agro Dijon, CNRS, Univ. Bourgogne, INRAE, Univ. Bourgogne Franche-Comté, F-21000 Dijon, France; 8 Agriculture and Agri-Food Canada, 960 Carling Avenue, Ottawa, Ontario, K1A 0C6, Canada

**Keywords:** *Acacia saligna*, nodules, *Rhizobium acaciae*, *Rhizobium leguminosarum *complex

## Abstract

Three bacterial strains, 1AS11^T^, 1AS12 and 1AS13, members of the new symbiovar salignae and isolated from root nodules of *Acacia saligna* grown in Tunisia, were characterized using a polyphasic approach. All three strains were assigned to the *Rhizobium leguminosarum* complex on the basis of *rrs* gene analysis. Phylogenetic analysis based on 1734 nucleotides of four concatenated housekeeping genes (*recA*, *atpD*, *glnII* and *gyrB*) showed that the three strains were distinct from known rhizobia species of the *R. leguminosarum* complex and clustered as a separate clade within this complex. Phylogenomic analysis of 92 up-to-date bacterial core genes confirmed the unique clade. The digital DNA–DNA hybridization and blast-based average nucleotide identity values for the three strains and phylogenetically related *Rhizobium* species ranged from 35.9 to 60.0% and 87.16 to 94.58 %, which were lower than the 70 and 96% species delineation thresholds, respectively. The G+C contents of the strains were 60.82–60.92 mol% and the major fatty acids (>4 %) were summed feature 8 (57.81 %; C_18 : 1_ ω7*c*) and C_18 : 1_ ω7*c* 11-methyl (13.24%). Strains 1AS11^T^, 1AS12 and 1AS13 could also be differentiated from their closest described species (*Rhizobium indicum*, *Rhizobium laguerreae* and *Rhizobium changzhiense*) by phenotypic and physiological properties as well as fatty acid content. Based on the phylogenetic, genomic, physiological, genotypic and chemotaxonomic data presented in this study, strains 1AS11^T^, 1AS12 and 1AS13 represent a new species within the genus *Rhizobium* and we propose the name *Rhizobium acaciae* sp. nov. The type strain is 1AS11^T^ (=DSM 113913^T^=ACCC 62388^T^).

## Introduction

*Acacia* is a multipurpose, fast-growing tree species of the family *Fabaceae*. This genus comprises over 1350 species that are distributed in the warm regions of the world, including Australia, the Americas, Africa and Asia [1]. *Acacia saligna* (Labill.) Wendl is an invasive, fast-growing woody tree that has been introduced to the Mediterranean coast in the Maghreb area including Tunisia for many different purposes, including revegetation of arid regions [2]. These plants enrich soil nitrogen in symbiotic association with various genera of rhizobia such as *Mesorhizobium*, *Rhizobium*, *Ensifer* and preferentially *Bradyrhizobium* [3]. In a recent study, Hsouna *et al*. [4] reported that *Acacia saligna* plants grown in Tunisian soils are promiscuous and were nodulated by fast growing organisms that could constitute putative new species within the genus *Rhizobium*. The 100 bacterial strains obtained in the study of Hsouna *et al*. [4] were characterized into three distinct ribotypes based on *rrs* PCR-RFLP analysis. Sequence analyses of *rrs* and four housekeeping genes (*recA*, *atpD*, *gyrB* and *glnII*) assigned 30 isolates to four putative new lineages of the genera *Rhizobium* and *Bradyrhizobium* and a single isolate to *Sinorhizobium meliloti*. One of these new lineages consisted of 10 isolates, including the representative strains 1AS11^T^, 1AS12 and 1AS13. Phylogenetic analysis of 1734-bp concatenated sequences of *recA*, *atpD*, *glnII* and *gyrB* clustered strains 1AS11^T^, 1AS12 and 1AS13 in a distinct clade within the *R. leguminosarum* complex (Rlc). The closest described species were *R. laguerreae* and *R. indicum* [4]. Interestingly, based on the divergence of *nod*A/*nod*C sequences and host range nodulation patterns used in previous study for symbiovar delineation [5,7], strains 1AS11^T^, 1AS12 and 1AS13 were classified as a new symbiovar named salignae [4] . The three strains effectively nodulated *A. saligna*, *A. salicina* and *Leucaena leucocephala*, while they failed to nodulate *Glycine max, Phaseolus vulgaris* and *Retama raetam*.

In the present study, the taxonomic status of strains 1AS11^T^, 1AS12 and 1AS13 was investigated using a polyphasic approach including genome-based analyses.

## Isolation and ecology

The three strains were isolated from root nodules of *A. saligna* grown in the locality of Borj Cedria in Tunisia [4]. Nodules were surface sterilized and bacteria were extracted following the standard procedures of Vincent [8]. Pure single colonies of the bacterial cultures were obtained by repetitive streaking on yeast–mannitol agar (YMA) as described previously [4]. Aliquots of the purified single colonies were preserved in 15 % (v/v) glycerol suspensions at −80 °C. The nodulation and nitrogen-fixation performance of the three novel strains was tested on various legume species.

## rrs, recA, atpD, gyrB and glnII phylogeny

Bacterial DNA was extracted from three isolates and used as template for 50 µl PCR reactions [4]. The *rrs* gene were sequenced using the primers fD1 and rD1 [9]. Amplification and sequencing of *recA*, *atpD*, *glnII* and *gyrB* genes were carried out according to the methods of Hsouna *et al*. [4]. PCR-amplified products were purified from agarose gels using the EZ-10 spin column DNA gel extraction Minipreps Kit (Bio Basic Canada Inc.) following the manufacturer’s instructions. Purified DNA fragments were sequenced at the Centre of Biotechnology, Borj-Cédria, Tunisia. The sequence similarities in the DNA databases were examined using the blast program (http://blast.ncbi.nlm.nih.gov/Blast.cgi). The *rrs* gene sequences were compared with NCBI reference sequences. Sequences were aligned using the ClustalW2 software (www.ebi.ac.uk/Tools/clustalw2/). Maximum-likelihood (ML) phylogenetic trees were inferred using the mega7 [10] using the Kimura two-parameter substitution model [11] with the pairwise deletion option [12]. Bootstrap analysis was based on 1000 re-samplings and values >50 % are shown on the phylogenetic trees.

**Fig. 1.**
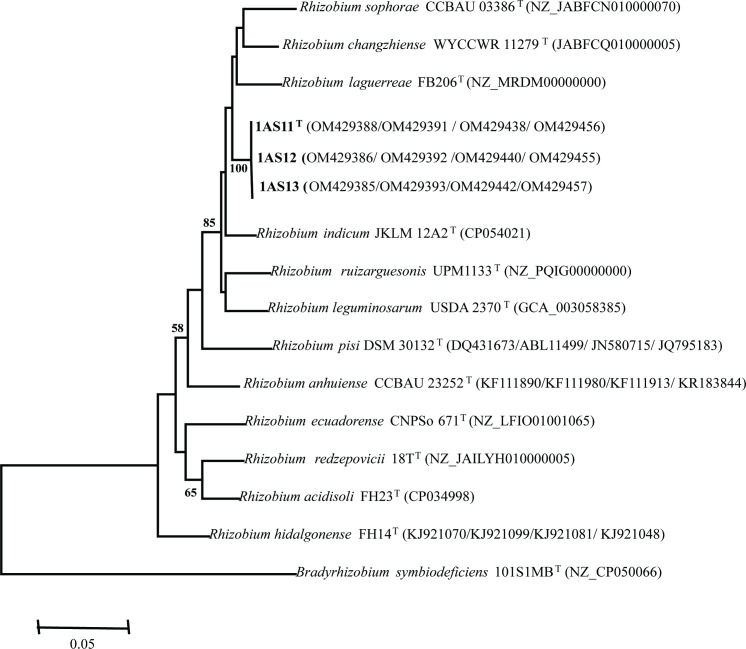
Maximum-Likelihood-based phylogenetic analysis of *recA-atpD-gyrB-glnII* concatenated gene sequences (1734 nucleotides). The three novel strains are in bold. Bootstrap values ≥50 are indicated for each node (1000 replicates). After species name, the strain designation is given followed by the NCBI accession number of the sequence used. The scale indicates the number of substitutions per site.

In the phylogenetic tree based on partial *rrs* gene sequences (1215 nt), all three new strains grouped together and showed 100 % similarity to *R. laguerreae*, *R. changzhiense*, *R. anhuiense*, *R. leguminosarum*, *R. ruizarguesonis*, *R. acidisoli*, *R. hidalgonense*, * R. indicum*, *R. sophorae* and *R. redzepovicii* type strains. Except for *R. anhuiense*, *R. acidisoli* and *R. hidalgonense*, these species were recently named to belong to the Rlc [13]. Fig. S1 (available in the online version of this article) shows an ML phylogenetic tree with the three strains of the proposed *R. acaciae* sp. nov. clustering with the type strains of the Rlc at a bootstrap value of 76 %. This suggests that the three strains of *R. acaciae* sp. nov. belong to the Rlc. Neighbour-joining and minimum-evolution algorithms were also used to reconstruct phylogenetic relationships and showed similar topologies (Figs S2 and S3). Given that, *rrs* phylogenies have been reported to show low-resolution power among closely related species [14,15], multilocus sequence analysis was performed using four housekeeping genes (*recA*, *atpD*, *gyrB* and *glnII*). Based on *recA-atpD-gyrB-glnII* concatenated sequences (1734 bp), the three novel strains were divergent from strains of type strains of previously described *Rhizobium* species of the Rlc group and the closest described species with were *R. indicum*, *R. laguerrea* and *R. changzhiense*. Based on the phylogenetic analysis of *recA-atpD-gyrB-glnII*, the three strains of *R. acaciae* sp. nov. clustered in a distinct clade (bootstrap of 100 %), an indication that these strains are unique (Fig. 1).

## Genomic relatedness and phylogenomics

Draft whole-genome sequences of the three new strains were generated using Illumina NovaSeq 6000 technology (Génome Québec, Montreal, Canada). The quality of the raw reads (2×150 bp) was checked using the FastQC algorithm [16]. *De novo* assembly was performed using Unicyler version 0.4.8 [17] and assessed using quast [18] as implemented in patric 3.6.12 [19,20]. Contigs of length <300 bp were discarded. Where required, assembled genome sequences were annotated using Prokka version 1.12 [21]. Table 1 shows the basic statistics of the sequenced draft genomes. For each of the strains, 33 112 583 raw reads were generated. After quality checks, the readsused in the assembling processed gave an average coverage depth of 1213.9×, 1314.8× and 1226.6× with contig counts of 84, 79 and 112, for strains 1AS11^T^, 1AS12 and 1AS13, respectively. The completeness of the genomes was 100 % with coarse and fine consistencies of 99.8–99.9% and 96.8–96.9 %, respectively, with a contamination rate of 0.5 % (CheckM algorithm) [22]. The largest contigs were 761366, 1 110 614 and 761 366 bases and had N_50_ values of 247715, 270 518 and 247 715 bases for strains 1AS11^T^, 1AS12 and 1AS13. The total lengths of the draft genomes were 8.01, 7.49 and 8.02 Mb, respectively, with G+C contents of 60.82–60.92 mol% (Table 1). Annotation reports of the genome sequences identified a total of 7714, 7127 and 7758 protein-coding sequences as well as 48 or 50 tRNAs, three rRNAs and one tmRNA (Table 1). The generated new draft whole-genome sequences were used to confirm the uniqueness of these strains relative to closest related *Rhizobium* species.

**Table 1.** Basic statistics of whole-genome sequences of the three strains of *Rhizobium acaciae* sp. nov, sequenced using Illumina NovaSeq 6000 technology

Description	1AS11^T^	1AS12	1AS13
Number of short reads (151 bp length)	33 112 583	33 112 583	33 112 583
Average coverage depth	1213.9×	1314.8×	1226.6×
Contig count	84	79	112
Largest contig (bp)	761 366	1 110 614	761 366
Total length (bp)	8 011 864	7 494 653	8 015 410
Contigs N_50_ (bp)	247 715	270 518	247 715
Contigs L_50_	12	9	12
Protein-encoding genes with functional assignment	5 037	4 743	5 036
Total protein-coding sequences (CDSs)	7 714	7 127	7 758
misc_RNA	75	67	75
Number of rRNA	3	3	3
Number of tRNA	48	50	48
Number of tmRNA	1	1	1
G+C content (mol%)	60.82	60.92	60.82

**Fig. 2.**
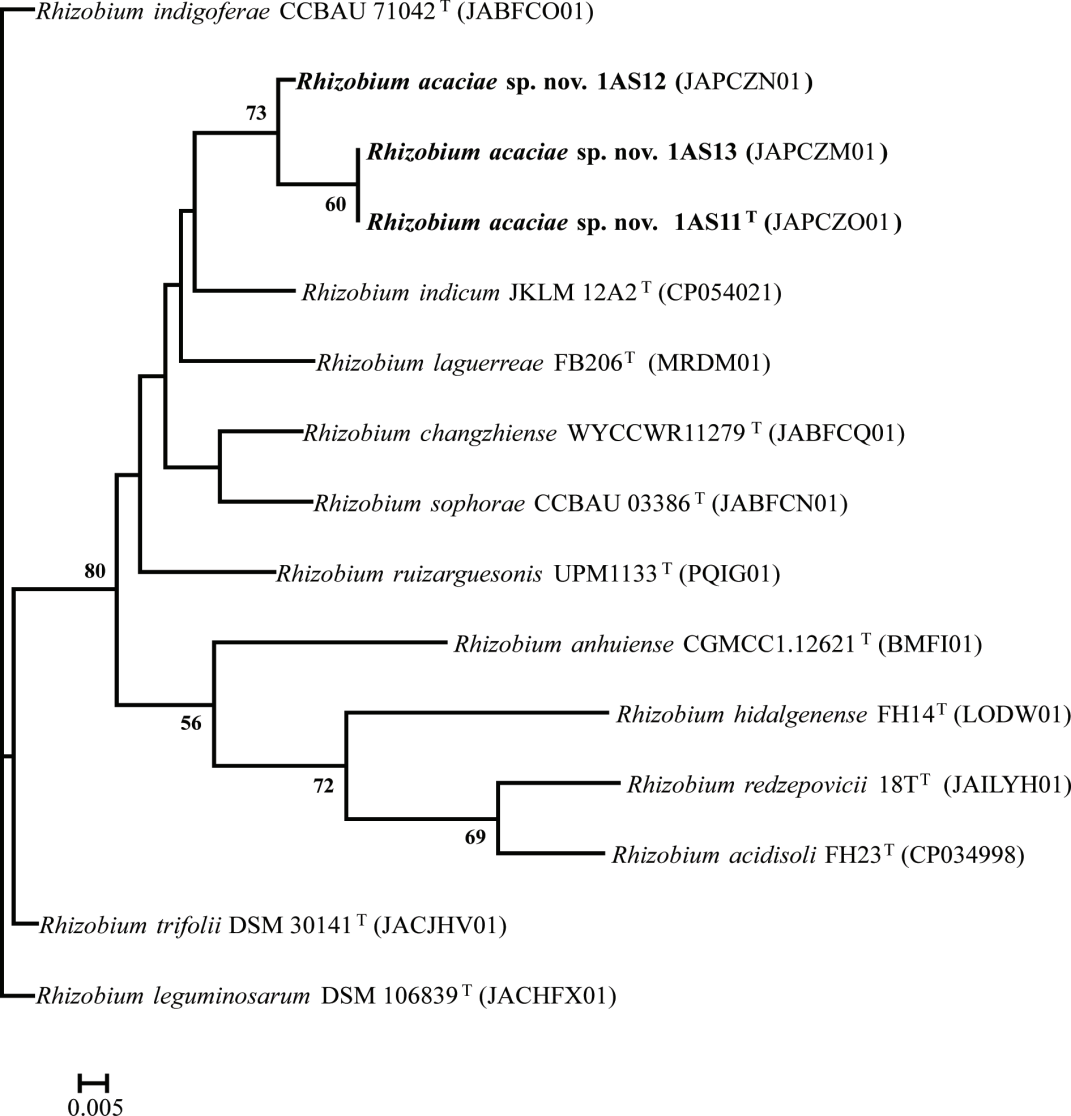
Unrooted maximum-likelihood phylogenetic tree based on UBCGs (concatenated alignment of 92 core genes) inferred using RAxML-ng with the GTR +CAT model. Percentage bootstrap values are given at branching points. Bar, 0.005 substitution per position.

Genome-sequence-based digital DNA–DNA hybridization (dDDH; GGDC 3.1 [23]), blast-based average nucleotide identity (ANIb; JSpecies [24]) and ANI (Fastani [25]) values were computed and compared to 12 validly published close *Rhizobium* species to validate the taxonomic position of strains. The three strains (1AS11^T^, 1AS12 and 1AS13) exhibited dDDH, ANIb and ANI values significantly above the species-level cut-offs of 70 and 96 %, validating that these three belong to the same *Rhizobium* species (Table 2). For example, strain 1AS11^T^ showed dDDH, ANI and ANIb values of 100, 99.99 and 99.97 % with strain 1AS13 (Table 2). Strain 1AS12 exhibited lower dDDH, ANb and ANI values with 1AS11^T^ but still significantly above the species-level cut-off (Table 2). The type strain of the proposed *R. acaciae* sp. nov, strain 1AS11^T^, had lower relatedness with the 12 closest species with values significantly below the cut-off values of 70 and 96 % for dDDH and ANIb and ANI, respectively (Table 2). *R. indicum* JKLM 12A2^T^ showed the highest dDDH of 60.0 % (cut-off, 70 %) and ANIb and ANI values of 94.58 and 94.95 % (cut-off, 96.00%) with strain 1AS11^T^ followed by *R. laguerreae* FB206^T^ with values of 56.10, 93.19 and 94.08 % (Table 2), all below the respective cut-off species delineation threshold. These data are consistent with the results of the *recA-atpD-gyrB-glnII* phylogenetic analysis, suggesting that strains 1AS11^T^, 1AS12 and 1AS13 constituted a novel species in the genus *Rhizobium*. The dDDH, ANIb and ANI results were corroborated by the phylogenomics tree [26,27] inferred using GBDP-derived intergenomic distances and computed on the Type (Strain) Genome Server (TyGS [28,29]) (Fig. S4). Also, the uniqueness of strains 1AS11^T^, 1AS12 and 1AS13 was validated by phylogenetic analysis of a concatenated alignment of 92 core genes as implemented in the up-to-date bacterial core gene (UBCG) tool [30] (Fig. 2). Both TyGS and UBCG pipelines generated phylogenomic trees that showed strains 1AS11^T^, 1AS12 and 1AS13 clustering uniquely from the closest known species within the genus *Rhizobium* (Fig. S4; Fig. 2). Based on these genome data and analyses, these new strains of the genus *Rhizobium* constitute a new species.

**Table 2.** Digital DNA–DNA hybridiziation (dDDH), average nucleotide identity (ANI) and blast-based average nucleotide identity (ANIb) values among *Rhizobium acaciae* sp. nov. AS11^T^ (type strain) and related *Rhizobium* species

*Rhizobium* species	dDDH	ANI	ANIb
*Rhizobium acaciae* sp. nov. 1AS11^T^ (JAPCZO01)	100	100	100
*Rhizobium acaciae* sp. nov. 1AS13 (JAPCZM01)	100 (0)	99.99	99.97
*Rhizobium acaciae* sp. nov. 1AS12 (JAPCZN01)	90.7 (0.0114)	98.84	98.72
*Rhizobium indicum* JKLM 12A2^T^ (CP054021)	60.0 (0.0515)	94.95	94.58
*Rhizobium laguerreae* FB 206^T^ (MRDM01)	56.1 (0.0588)	94.08	93.19
*Rhizobium changzhiense* WYCCWR 11279^T^(JABFCQ01)	53.8 (0.0634)	93.68	93.08
*Rhizobium sophorae* CCBAU 03386^T^ (JABFCN01)	53.2 (0.0645)	93.59	92.50
*Rhizobium ruizarguesonis* UPM 1133T (PQIG01)	52.7 (0.0656)	93,52	92,27
*Rhizobium leguminosarum* DSM 106839^T^ (JACHFX01)	49.1 (0.0737)	92.67	91.34
*Rhizobium indigoferae* CCBAU 71042 (JABFCO01)	49.0 (0.0740)	92.66	91.57
*Rhizobium trifolii* DSM 30141^T^ (JACJHV01)	49.2 (0.0736)	92.60	91.48
*Rhizobium anhuiense* CGMCC 112621^T^ (BMFI01)	42.5 (0.0915)	91.07	89.97
*Rhizobium acidisoli* FH23^T^ (CP034998)	37.3 (0.1090)	89.50	88.79
*Rhizobium redzepovicii* 18T^T^ (JAILYH01)	36.5 (0.1123)	88.91	87.60
*Rhizobium hidalgenense* FH14^T^ (LODW01)	35.9 (0.1146)	88.68	87.16

**Fig. 3.**
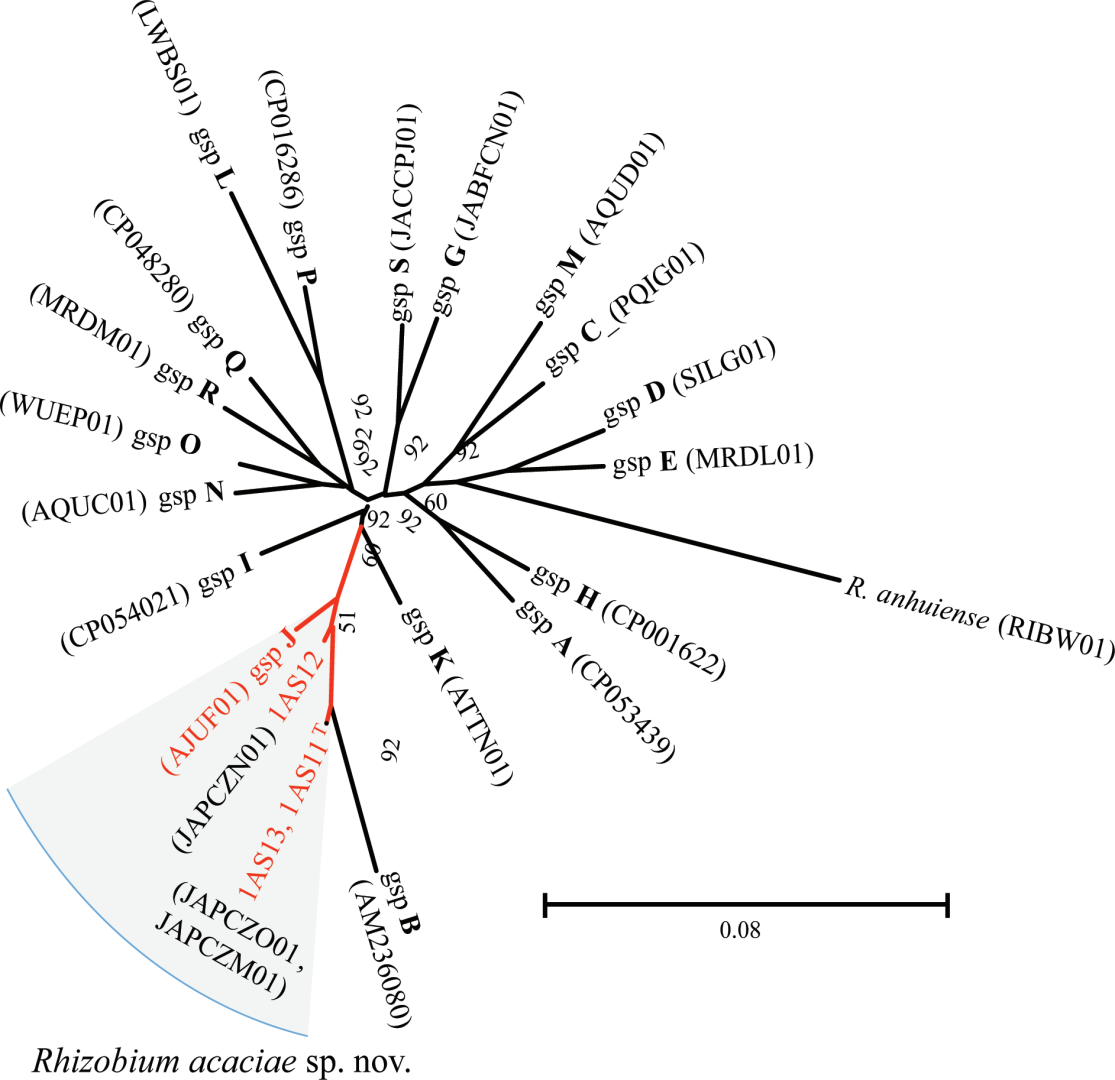
Maximum-likelihood phylogenetic tree based on UBCGs (concatenated alignment of 92 core genes) of strains of *Rhizobium acaciae* sp. nov. and representatives of the 18 genomospecies proposed by Young *et al*. [13]. Tree was inferred using RAxML-ng with the GTR +CAT model. Percentage bootstrap values are given at branching points. Strains of *R. acaciae* sp. nov. are affiliated with genomospecies j. Letters in bold denote the genomospecies proposed by Young *et al*. [13] .

In addition, the whole genome sequences generated in this study were used to locate the proposed new species within the 18 genomospecies (gsp) groupings according to Young *et al*. [13] . The UBCG tool was used to implement an ML phylogenomics tree of strains 1AS11^T^, 1AS12 and 1AS13 and the proposed representatives of the genomospecies of Young *et al*. [13]. Fig. 3 shows an unrooted tree of the *R. acaciae* strains clustering closely with the representative strain of gsp J. The gsp J was represented by two strains WSM1455 (AJUF01) and WSM1481 (AQUM01) and these strains were classified as unique [13] but deposited in GenBank as *Rhizobium leguminosarum* bv. viciae. Interestingly, a taxonomy check using GenBank’s algorithm flagged these two strains as “inconclusive “ to be classified as *R. leguminosarum*. These two unique strains, WSM1455 (AJUF01) and WSM1481 (AQUM01), exhibited dDDH and ANI values of 83.10 and 98.07 % with the type strain of the proposed *R. acaciae* 1AS11^T^. These values are significantly above the species-level cut-off of 70 and 96 %, respectively, suggesting that strains WSM1455 and WSM1481 are genetically valid members of the proposed new species, *R. acaciae* sp. nov. Both WSM1481 and WSM1455 were isolated from roots of *Vicia faba* plants in the Greek island of Mykonos (GOLD database; https://gold.jgi.doe.gov/organism?id=Go0007354). This suggests that members of the proposed new species, *R. acaciae* sp. nov., might have preference for plant species of the family *Fabaceae*, from cultivated legumes (broad beans) to small wild trees, e.g. golden wreath wattle (*Acacia saligna*).

## Physiology and chemotaxonomy

Colonies of strains 1AS11^T^, 1AS12 and 1AS13, as examined on YMA, were typical rhizobial-like translucent, cream-white and convex. The morphology of the bacterial species was examined with an atomic force microscope (FlexAFM Nanosurf AG) operated in the tapping imaging mode in phase contrast, at room temperature, using a CoreAFM Microscope, Nanosurf Isostage 300. Cells were 0.4–0.5×1–2 µm in size (Fig. S5). The phenotypic and physiological features of the novel isolates were determined and compared with their most related type strains. The characteristics tested included the use of sole carbon and nitrogen sources, tolerance to NaCl, pH, resistance to antibiotics and fatty methyl ester content. Biochemical characteristics, enzyme activities and utilization of carbon sources were performed using the API ZYM systems (07584D and 25 200, bioMérieux) and the Biolog GEN III system following the manufacturers’ instructions. Antibiotic resistance testing was carried out on Yeast extract Mannitol (YEM) plates supplemented with five concentrations of each antibiotic (5, 10, 50 80 and 100 µg ml^−1^). The antibiotics used were streptomycin, kanamycin, ampicillin, neomycin, erythromycin, chloramphenicol and tetracycline. Tolerance to NaCl, pH and temperature was checked in liquid YEM as previously reported [31] .The optimum pH for growth was assessed on YMA medium plates adjusted to pH 4–10 using 1 M HCl (pH 4.0 and 5.0) or 1M NaOH (pH 7–10 prior to autoclaving).

Carbon substrate characteristics (Table 3) shared by all three strains of *R. acaciae* sp. nov. that differed from at least one of their closest related type strains were the ability to utilize trehalose, turanose, raffinose, lactose, melibiose, methyl β-d-glucoside, *N*-acetyl-d-glucosamine, *N*-acetyl-β-d-mannosamine, α-d-glucose, d-mannose, d-galactose, d-fucose, l-rhamnose, arabitol and *myo*-inositol. In addition, strains of *R. acaciae* were able to use l-glutamic acid, l-histidine and l-pyroglutamic acid. Other characteristics shown by the strains of the novel species that differed from at least one of these closest type strains were their weak positive enzymatic reaction for cystinol-arylamidase, α-mannosidase and β-fucosidase and negative reactions for *N*-acetyl-β-glucosaminase. *Rhizobium acaciae* sp. nov. strains were resistant to kanamycin (10 µg ml^−1^) and streptomycin (10 µg ml^−1^) but sensitive to lincomycin and rifamycin. Fatty acid methyl esters were analysed using the Microbial Identification System (midi) and the Microbial Identification software package (Sherlock version 6.1; midi database, TSBA6). The fatty acid composition and abundance varied between 1AS11^T^ and closely related type strains of *Rhizobium* species (Table 4). Indeed, the amount of C_18 : 1_ ω7*c* 11-methyl in strain 1AS11^T^ was higher than that in strains FB206^T^ of *R. laguerreae* and JKLM12A2^T^ of *R. indicum*. Also, we detected small amounts of C_15 : 0_ 3OH (0.27 %), C_17 : 0_ (0.78 %), C_17 : 1_ anteiso ω9*c* (0.32 %), C_17 : 1_ ω8*c* (0.26 %), C_17 : 1_ ω6*c* (0,1 %), C_17 : 0_ 3OH (0.16 %), C_20 : 1_ ω7*c* (0.45 %) and C_20 : 2_ ω6,9*c* (0.17 %) that were absent in FB206^T^ and JKLM12A2^T^.

**Table 3.** Phenotypic results on Biolog GENIII MicroPlates Strains: 1AS11^T^; 2, 1AS12; 3, 1AS13; 4, *Rhizobium indicum* JKLM 12A2^T^; 5, *Rhizobium laguerreae* FB206^T^; 6, *Rhizobium changzhiense* WYCCWR 11279^T^. +, Positive; −, negative; w, weakly positive.

	*Rhizobium acaciae* sp. nov.					
Biolog substrate	1	2	3	4	5	6
Trehalose	+	+	+	w	−	−
Sucrose	−	−	−	+	−	−
Turanose	+	+	+	w	−	−
Raffinose	+	+	+	−	−	−
Lactose	+	+	+	−	−	−
Melibiose	+	+	+	−	+	−
Methyl β-d-glucoside	+	+	+	−	+	+
*N*-Acetyl-d-glucosamine	+	+	+	−	+	+
*N*-Acetyl-β-d-mannosamine	+	+	+	−	+	−
*N*-Acetyl-d-galactosamine	−	−	−	−	+	−
N-Acetyl neuraminic acid	−	−	−	−	−	+
α-d-Glucose	+	+	+	−	−	−
d-Mannose	+	+	+	−	−	−
d-Galactose	+	+	+	−	+	+
d-Fucose	+	+	+	−	+	+
l-Rhamnose	+	+	+	−	−	+
dArabitol	+	+	+	−	−	+
*myo*-Inositol	+	+	+	−	−	+
Glycerol	+	+	+	−	+	+
d-Fructose-6-PO_4_	−	−	−	+	+	−
l-Glutamic acid	+	+	+	−	+	+
l-Histidine	+	+	+	−	−	+
l-Pyroglutamic acid	+	+	+	−	+	−
d-Galacturonic acid	−	−	−	+	+	−
d-Gluconic acid	+	+	+	−	+	+
d-Glucuronic acid	−	−	−	+	+	−
Mucic acid	−	−	−	−	−	+
Quinic acid	−	−	−	−	−	+
Methyl pyruvate	+	+	+	−	−	−
l-Lactic acid	−	−	−	−	−	+
d-Malic acid	−	−	−	−	−	+
l-Malic acid	−	−	−	−	+	w
Tween 40	−	−	−	−	w	+
α-Hydroxy-butyric acid	−	−	−	−	−	+
β-Hydroxy-d,l butyric acid	−	−	−	−	+	+
α-Keto-butyric acid	−	−	−	−	−	+
Acetoacetic acid	−	−	−	−	−	+
Propionic acid	−	−	−	−	−	+
Acetic acid	−	−	−	w	−	+
Formic acid	−	−	−	−	−	+
**Chemical sensitivity**						
pH 6	+	+	+	−	−	−
pH 5	+	+	+	−	−	−
NaCl 1%	−	−	−	−	+	−
**Antbiotic resistance**						
Rifamycin	−	−	−	−	−	+
Lincomycin	−	−	−	−	−	+
Kanamycin	+	+	+	NA	−	−
Streptomycin	+	+	+	NA	−	−
**API ZYM**						
Alkaline phosphomonoesterase	−	−	−	−	−	w
Esterase	−	−	−	NA	−	w
Cystinol-arylamidase	w	w	w	NA	+	+
*N*-Acetyl-β-glucosaminase	−	−	−	NA	−	+
α-Mannosidase	w	w	w	NA	+	+
β-Fucosidase	w	w	w	NA	+	+

**Table 4.** Fatty acid composition (%) of 1AS11^T^ and its related *Rhizobium* type strains Strains: 1, 1AS11^T^; 2, *Rhizobium indicum* JKLM 12A2^T^; 3, *Rhizobium laguerreae* FB206^T^; 4, *Rhizobium changzhiense* WYCCWR 11279^T^. –, Not detected.

Fatty acid	1	2	4	4
C_15 : 0_ 3OH	0.27	–	–	–
C_17 : 0_	0.78	–	–	–
C_17 : 1_ ω8*c*	0.26	–	–	–
C_17 : 1_ ω6*c*	0.1	–	–	–
C_17 : 0_ 3OH	0.16	–	–	–
C_17 : 1_ anteisoω9*c*	0.32	–	–	–
C_18 : 1 _ω9*c*	0.6	–	–	0.5
C_18 : 1_ ω7*c* 11-methyl	13.24	1.8	1.9	–
C_20 : 1_ ω7*c*	0.45	–	–	–
C_20 : 2_ ω6,9*c*	0.17	–	–	–
Summed feature 8*	57.81	68	69.3	74.9

*Summed feature 8: C_18 : 1_ ω7*c*.

## Description of *Rhizobium acaciae* sp. nov.

*Rhizobium acaciae* (a.ca'ci.ae. L. gen. n. *acaciae*, of the plant genus *Acacia* the host plant from which the novel species was isolated).

Cells of *R. acaciae* are Gram-stain-negative, non-spore forming rods. Colony morphology, as examined on YMA, was typical rhizobial-like translucent, cream-white and convex after 2–3 days of incubation at 28 °C. All strains grew at 16–37 °C (optimum, 28 °C) and pH 6–10 (optimum, pH 7). Strains 1AS11^T^, 1AS12 and 1AS13 were not able to grow at NaCl concentrations higher than 0.6 %. Detailed phenotypic and biochemical data for the *R. acaciae* strains are given in Table 3. The strains can be distinguished from their most closely related *Rhizobium* species by their ability to use trehalose, turanose, raffinose, lactose, melibiose, methyl β-d-glucoside, *N*-acetyl-d-glucosamine, *N*-acetyl-β-d-mannosamine, α-d-glucose, d-mannose, d-galactose, fucose, l-rhamnose, d-arabitol and *myo*-inositol as sole carbon sources; the strains showed weak positive enzymatic reactions for cystinol-arylamidase, α-mannosidase and β-fucosidase and negative reactions for *N*-acetyl-β-glucosaminase. Strains are resistant to 10 µg ml^−1^ kanamycin and 10 µg ml^−1^ streptomycin, but sensitive to lincomycin and rifamycin. The major fatty acid of the type strain 1AS11^T^ is C_18 : 1_ ω7*c* with 57.81 % content.

The type strain of *Rhizobium acaciae* sp. nov. is 1AS11^T^ (=DSM 113913^T^=ACCC 62388^T^), which was isolated from root nodules of *Acacia saligna* grown in Borj Cedria, a city in Ben Arous governorate, Tunisia. The G+C content of 1AS11^T^ is 60.82 mol% and the genome size is approximately 8.01 Mb.

## supplementary material

10.1099/ijsem.0.005900 Uncited Fig. S1.

## Abbreviations

ANI: Average Nucleotide Identity
dDDH: Digital DNA-DNA hybridization
YMA: Yeast Mannitol Agar
