# Supplementary material for: Rhizobium acaciae sp. nov., a new nitrogen-fixing symbiovar isolated from root nodules of Acacia saligna in Tunisia
Source: Int J Syst Evol Microbiol. 2023 May 18;73(5):005900. doi: 10.1099/ijsem.0.005900 (PMC11426921; doi:10.1099/ijsem.0.005900)
Supplement: Uncited Fig. S1. [file ijsem-73-05900-s001.pdf]

**Supplementary materials**

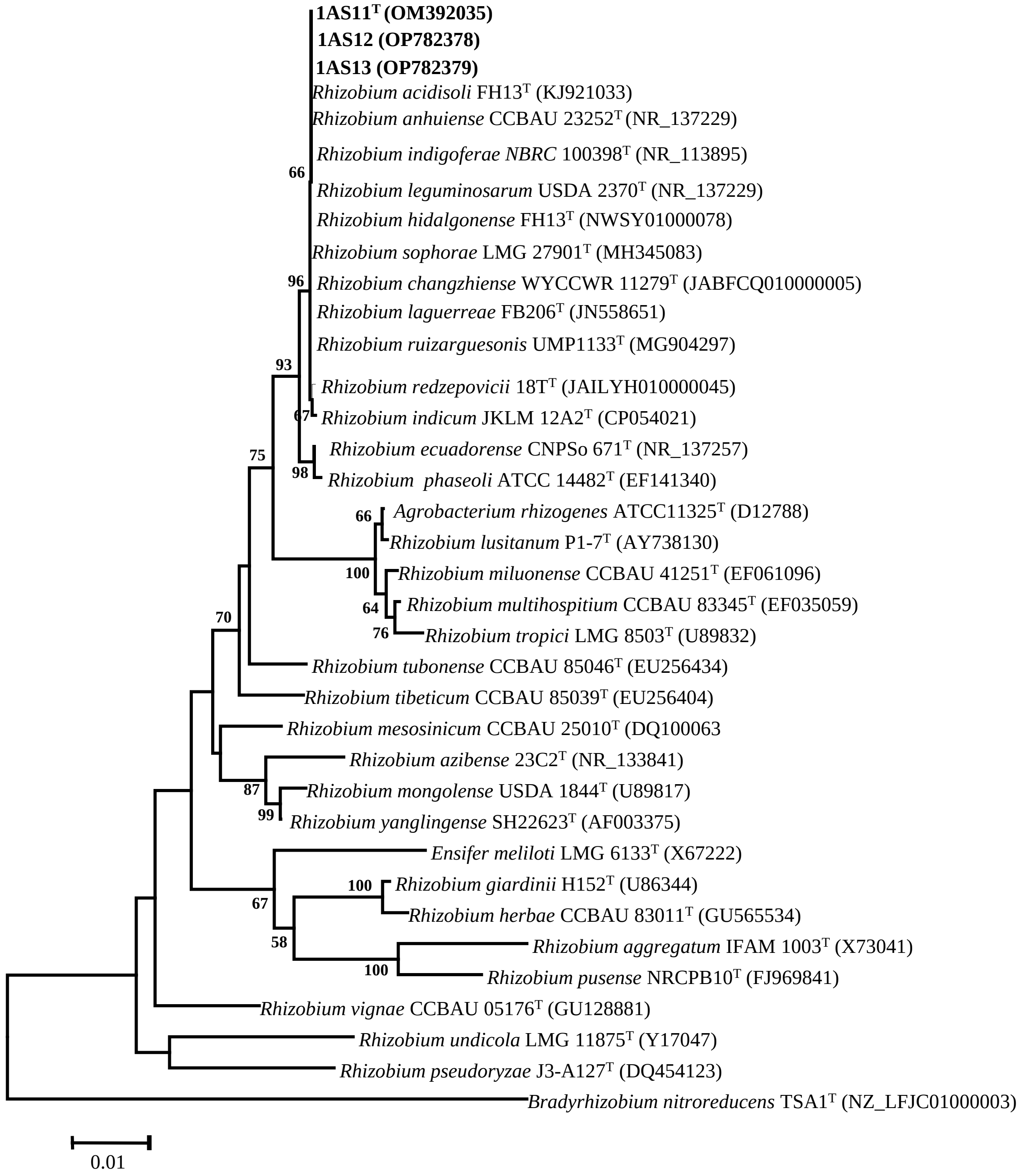


**Figure S1**: Phylogenetic tree of *rrs* sequences (1215 nucleotides) based on the maximum likelihood method. The three novel strains are in bold. Bootstrap values ≥50 are indicated for each node (1000 replicates). After species name, the strain designation followed by the NCBI accession number of the sequence used. The scale indicates the number of substitutions per site.

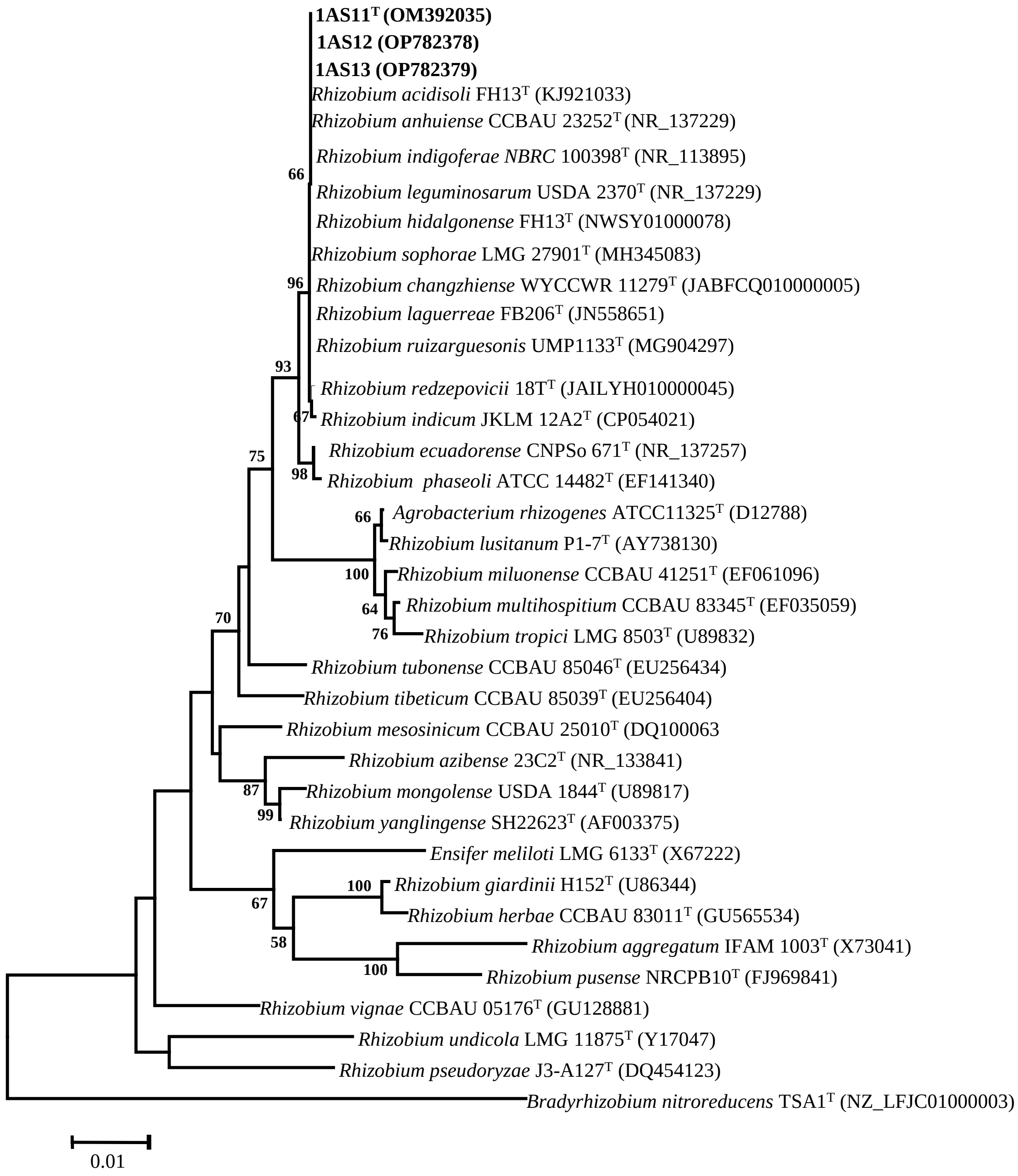


**Figure S2**: Phylogenetic tree of *rrs* sequences (1215 nucleotides) based on the Neighbor-Joining Method. Representative strains trapped by *Acacia saligna* are in bold. Bootstrap values ≥50 are indicated for each node (1000 replicates). After species name, the strain designation followed by the NCBI accession number of the sequence used. The scale indicates the number of substitutions per site.

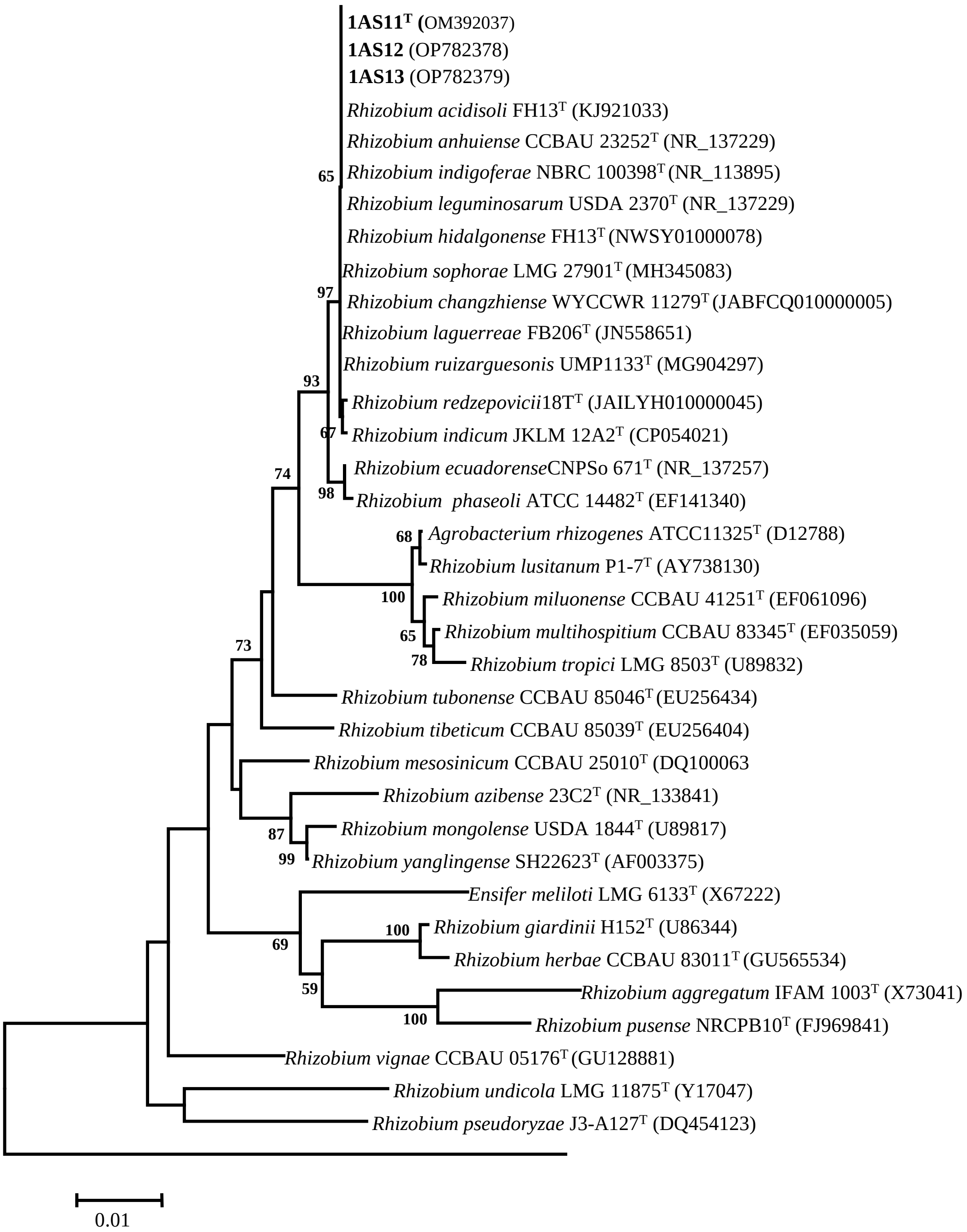


**Figure S3**: Phylogenetic tree of *rrs* sequences (1215 nucleotides) based on the minimum evolution Method. Representative strains trapped by *Acacia saligna* are in bold. Bootstrap values ≥50 are indicated for each node (1000 replicates). After species name, the strain designation followed by the NCBI accession number of the sequence used. The scale indicates the number of substitutions per site.

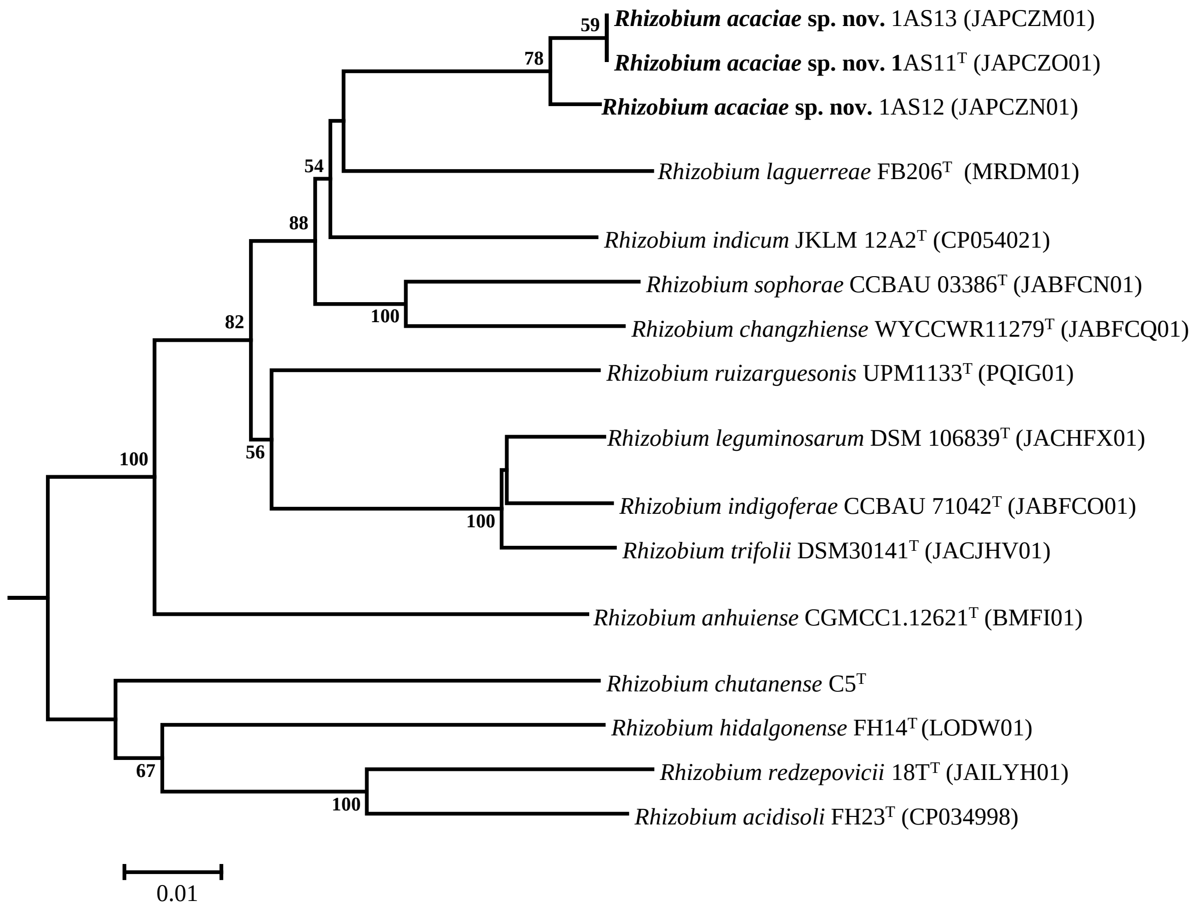


**Figure S4:** Tree inferred with FastME 2.1.6.1 [26] from GBDP distances calculated from genome sequences. The branch lengths are scaled in terms of GBDP distance formula d5. The numbers above branches are GBDP pseudo-bootstrap support values > 60 % from 100 replications, with an average branch support of 68.4 %. The tree was rooted at the midpoint (Farris 1972).

**Figure S5**

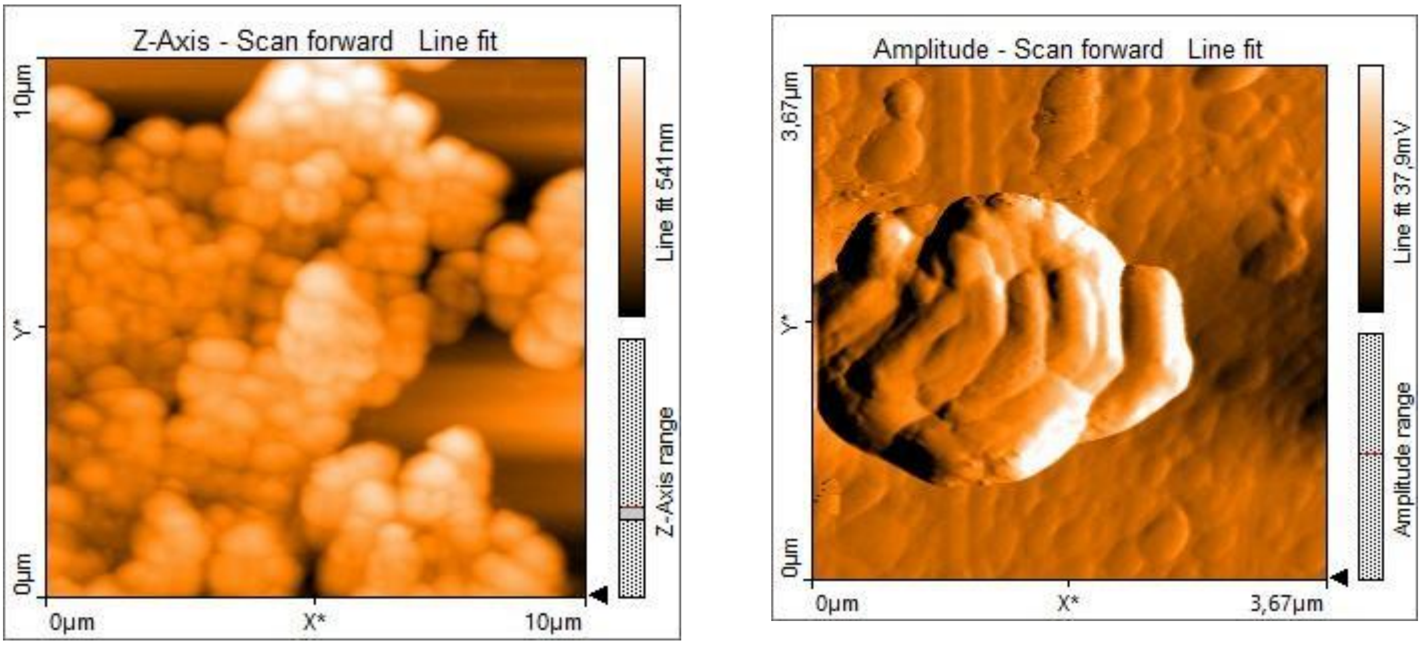


**Figure S5.** AFM images of the type strain 1AS11$^T$ of *R. acaciae* sp. nov
